# Embedding planetary health and sustainability in dental education: a community-engaged training model from Kenya

**DOI:** 10.3389/froh.2026.1878019

**Published:** 2026-09-04

**Authors:** James Johnstone Nyachio, Gladys Mwango Akama, Ochiba Lukandu

**Affiliations:** 1 Amref International University (AMIU), Candidate MPH in Epidemiology, Nairobi, Kenya; 2 Department of Community, Preventive Dentistry and Periodontolgy, School of Dentistry, College of Health Sciences, Moi University, Eldoret, Kenya; 3 School of Dentistry, College of Health Sciences, Moi University, Eldoret, Kenya

**Keywords:** climate change, COBES, community-based education, dental education, global health education, Kenya, LMIC, oral health systems

## Abstract

Climate change and environmental degradation are reshaping health systems in ways that the dental profession can no longer afford to ignore. Oral health is increasingly positioned as both a victim of environmental disruption and a contributor to it. However, despite growing momentum in medicine and public health, dental education has remained largely disconnected from climate–health thinking and sustainability frameworks. This manuscript examines how planetary health principles can be meaningfully integrated into dental education through community-engaged learning. Drawing on the Community-Based Education and Service programme at Moi University College of Health Sciences in Kenya, we present a conceptual framework built on four interrelated components: curriculum reform, community-based training, sustainable clinical practice, and interdisciplinary collaboration. This approach supports prevention-focused care, develops systems thinking, and produces graduates who are equipped to engage with environmental determinants of health. This approach is designed to be scalable and adaptable, with particular relevance to low- and middle-income countries. It connects directly to global priorities, including the Sustainable Development Goals, Universal Health Coverage, and the WHO Global Oral Health Action Plan 2023–2030.

## 1 Introduction

Planetary health, as a field, rests on a clear and sobering premise: Human health cannot be sustained if we continue to erode the natural systems that support it (1, 2). Climate change, biodiversity loss, and environmental degradation are reshaping disease patterns and deepening health inequities across the globe (3, 4). These are not abstract, future-facing concerns; they are already changing how oral diseases present, how they are distributed, and how they are managed.

Oral diseases affect an estimated 3.5 billion people globally, making them among the most prevalent non-communicable conditions worldwide (5, 6). Their burden is shaped not only by biological factors but also by environmental, social, and economic forces. Climate-related disruptions such as food insecurity, water scarcity, and pollution worsen oral health risks while simultaneously destabilising the infrastructure that communities depend on for care (7, 8). A scoping review by Bhadauria and colleagues (9) confirmed direct and indirect pathways through which environmental change reshapes oral health outcomes.

In spite of these connections, dental education has remained anchored in biomedical and clinical competencies. Evidence of this gap is consistent across contexts. Joury et al. (10) found that two leading dental schools in the UK and the US did not formally teach environmental sustainability. Gershberg et al. (11) reported that while over 80% of US dental students viewed sustainability as important, fewer than 5% had encountered it in their curricula. A UK and Ireland survey found 56% of dental schools currently do not teach environmental sustainability (12). Martin et al. (13) identified limited professional awareness as the primary barrier to change.

Sub-Saharan Africa presents an especially important context for this conversation. Kenya carries a high oral disease burden alongside constrained dental workforce capacity, rapidly urbanising communities, and growing exposure to droughts, flooding, and food system disruption (14, 15). In this setting, integrating planetary health into dental training is not an aspirational add-on; it is a systems-level imperative that oral health education can no longer defer.

## 2 Planetary health and oral health

The relationship between environmental systems and oral health is closer than dental curricula typically acknowledge. Water availability, food systems, climate variability, and exposure to pollutants all shape oral disease across the life course in ways that are both significant and underappreciated (1, 16). Understanding these pathways is foundational to any meaningful reform of how we train oral health professionals.

Water scarcity limits oral hygiene and preventive care in ways that fall hardest on rural and low-income communities (7). Changes in food systems are accelerating reliance on ultra-processed, sugar-heavy diets, driving up caries rates, particularly in children (5, 17). Air pollution and environmental toxicants have been linked to oral mucosal pathology, periodontal disease, and enamel defects (18). When extreme weather strikes and health infrastructure collapses, dental services are among the first disrupted.

Fisher et al. (19) proposed an integrated model situating oral disease within planetary boundaries, arguing that biodiversity loss, climate change, and pollution act synergistically on oral health. Their work calls for planet-centric critical thinking in oral health education and practice. This is directly relevant to the training framework stated in this paper, which seeks to move dental education towards a systems-oriented understanding of health rooted in real community contexts (6, 20).

## 3 Sustainability in dentistry

Dentistry leaves a larger environmental footprint than many within the profession acknowledge. Energy consumption, biomedical waste, mercury use in amalgam, and dependence on single-use disposables all contribute measurably to the environmental burden of healthcare (21, 22). As health systems globally move towards sustainability, dentistry must engage with this agenda directly, rather than waiting for external pressure to force the conversation.

The FDI World Dental Federation has taken a clear leadership stance. Its Policy Statement on Sustainability in Dentistry (47) calls for integrating the Sustainable Development Goals (SDG) into daily dental practice and supporting a shift towards a green economy. Building on this, a three-wave Delphi process culminated in a Joint Stakeholder Consensus Statement released at the FDI Sustainability in Dentistry Summit in March 2022 (23). In December 2023, the FDI launched a Massive Open Online Course on Sustainability in Dentistry to equip dental teams and students with practical knowledge for environmentally responsible practice (24).

Martin and Mulligan (25) argued that environmental sustainability through good-quality oral healthcare is achievable and intrinsically aligned with prevention-oriented models of care. A scoping review (13) identified eight interconnected themes—including waste management, procurement, and education—and confirmed that limited educational provision remains a primary barrier. Preventive approaches are themselves low-carbon: A tooth not decayed generates no drill, no suction, no single-use consumable, and no clinical waste. That alignment between clinical excellence and environmental responsibility is a powerful argument for re-centring prevention in dental training.

## 4 Community-based education in dental training

Community-based education offers something that classroom and clinical training alone cannot: direct exposure to the social, environmental, and economic realities that shape health. The Community-Based Education and Service (COBES) model at Moi University College of Health Sciences is one of East Africa's most established examples of this approach. Students engage in extended rotations within rural and underserved communities, working alongside local health systems, community health workers, and community members (26, 27).

In these settings, students encounter health challenges in their full complexity. They see how access to clean water shapes oral hygiene behaviours, how food insecurity translates into caries risk, and how communities navigate climate-related agricultural disruption alongside dental disease. These conditions are not peripheral to clinical learning; they are the curriculum. Structuring learning around these realities moves oral health training from a technical competency model towards a systems-oriented, equity-informed professional formation.

COBES creates a largely untapped opportunity to embed planetary health concepts in dental training. Communities served under COBES face compound vulnerabilities: constrained access to clean water, food insecurity, and disrupted pathways to oral health services. Formal integration of environmental health assessments into student placements would make these vulnerabilities explicit objects of clinical reasoning, rather than assumed backdrops. This is where concepts like health equity, environmental determinants, and sustainability stop being abstract lecture content and become lived professional experience.

## 5 Integrating planetary health into dental education: a proposed framework

We propose a framework for integrating planetary health into dental education through four interrelated components, designed to work within existing training architectures and adaptable across diverse low- and middle-income country (LMIC) contexts. This framework builds explicitly on prior contributions, including Duane et al. (28), who mapped current practice in embedding environmental sustainability within dental curricula internationally, and Field et al. (29), who established competency-based learning outcomes through an Association for Dental Education in Europe (ADEE) Special Interest Group consensus process (45). We extend these contributions by grounding implementation within a community-engaged, LMIC training context that prior models have not adequately addressed.

### 5.1 Curriculum reform

Planetary health, environmental determinants of oral health, and sustainability must enter core dental programmes as structured, formally assessed, and progressively developed competencies. This is not about adding a standalone module at the margins of training. It requires reviewing national dental training standards and accreditation frameworks to create genuine curricular space across all years of study. Foundational sciences should incorporate environmental epidemiology and social determinants. Clinical years should address sustainable practice, responsible prescribing, and waste management. Final-year training should engage students with policy, advocacy, and systems-level change (44).

Field et al. (29) proposed a suite of learning outcomes for environmental sustainability within the Graduating European Dentist (GED) curriculum framework, formally incorporated into the GED framework in 2023 (30). These outcomes span knowledge, attitudes, and clinical decision-making competencies, structured progressively across all years of training. While developed in a European context, they provide a solid reference that African dental schools and regulatory bodies can adapt with appropriate contextualisation.

Evidence now exists that structured teaching produces measurable results. Dixon et al. (31) implemented a comprehensive 5-year longitudinal strategy embedding environmental sustainability across all years of an undergraduate dental curriculum. Their study demonstrated significant positive changes in student awareness, attitudes, and knowledge, providing the first empirical evidence that sustained, curriculum-wide reform can achieve meaningful educational outcomes in this domain. Their companion seven-component iterative curriculum development model, grounded in Kern's model and adapted for dental education, offers a practical validated pathway for schools undertaking similar reform (46).

Even shorter interventions can be effective when designed well. Baird et al. (32) evaluated a two-part interactive workshop combining didactic sessions with group problem-solving and student-led presentations. Pre- and post-workshop surveys showed significant improvements in student confidence and knowledge across sustainability domains. A global scoping review by Bamedhaf et al. (33) confirmed that structured, outcome-aligned teaching consistently outperforms passive or unstructured approaches regardless of institutional setting. The evidence is clear: How sustainability is taught matters as much as whether it is included.

### 5.2 Community-based learning

Competency-Based Education (CBE) platforms such as COBES must be deliberately leveraged to link theoretical planetary health knowledge with real-world environmental health challenges. Structured learning objectives should explicitly name environmental determinants as targets of clinical reasoning and health promotion. Students should conduct environmental health assessments alongside oral health screenings during community placements, identifying risk factors such as water quality, sanitation access, and food environment. These assessments should be formally evaluated as part of placement requirements. This transforms community engagement from an experiential enrichment into a rigorous vehicle for sustainability competency development.

### 5.3 Sustainable clinical practice

Training environments must model the environmentally responsible behaviours they expect graduates to adopt. This includes adopting digital radiography to reduce chemical waste, investing in efficient autoclave systems and proper waste segregation, and implementing procurement policies that favour biocompatible and low-impact materials. Clinical culture must visibly prioritise prevention and minimal intervention. Faculty and supervisors need to be equipped and incentivised to embed sustainability into clinical teaching and assessment, not as an optional add-on, but as a dimension of clinical quality. Duane and Fennell-Wells (34) have published evidence-aligned clinical guidelines for environmental sustainability in dentistry, which dental schools can adopt as a practical framework for standardising sustainable training environments.

### 5.4 Interdisciplinary collaboration

Planetary health does not respect disciplinary boundaries. Genuine engagement requires dental graduates who can work effectively alongside public health professionals, environmental scientists, nurses, physicians, social workers, and policy practitioners. Joint teaching, interdisciplinary project work, and shared community placements build precisely these collaborative competencies. In the Kenyan context, formal integration with existing One Health platforms and national climate–health policy frameworks offers a practical and institutionally legitimate pathway for achieving this.

The barriers to interdisciplinary collaboration in sub-Saharan African health education are well documented and must be taken seriously. A systematic review by Kitema et al. (35) identified professional siloing, institutional resistance, limited faculty capacity in interdisciplinary pedagogy, and inconsistent accreditation support as persistent structural challenges. Delawala et al. (36) further emphasised that interprofessional education integration is context-dependent and requires leadership commitment, early stakeholder engagement, and piloting before scale-up. Addressing these barriers requires formal faculty development, phased implementation starting with shared community placements, and explicit alignment with national health workforce and One Health policy frameworks.

## 6 Conceptual framework

**Figure 1:**
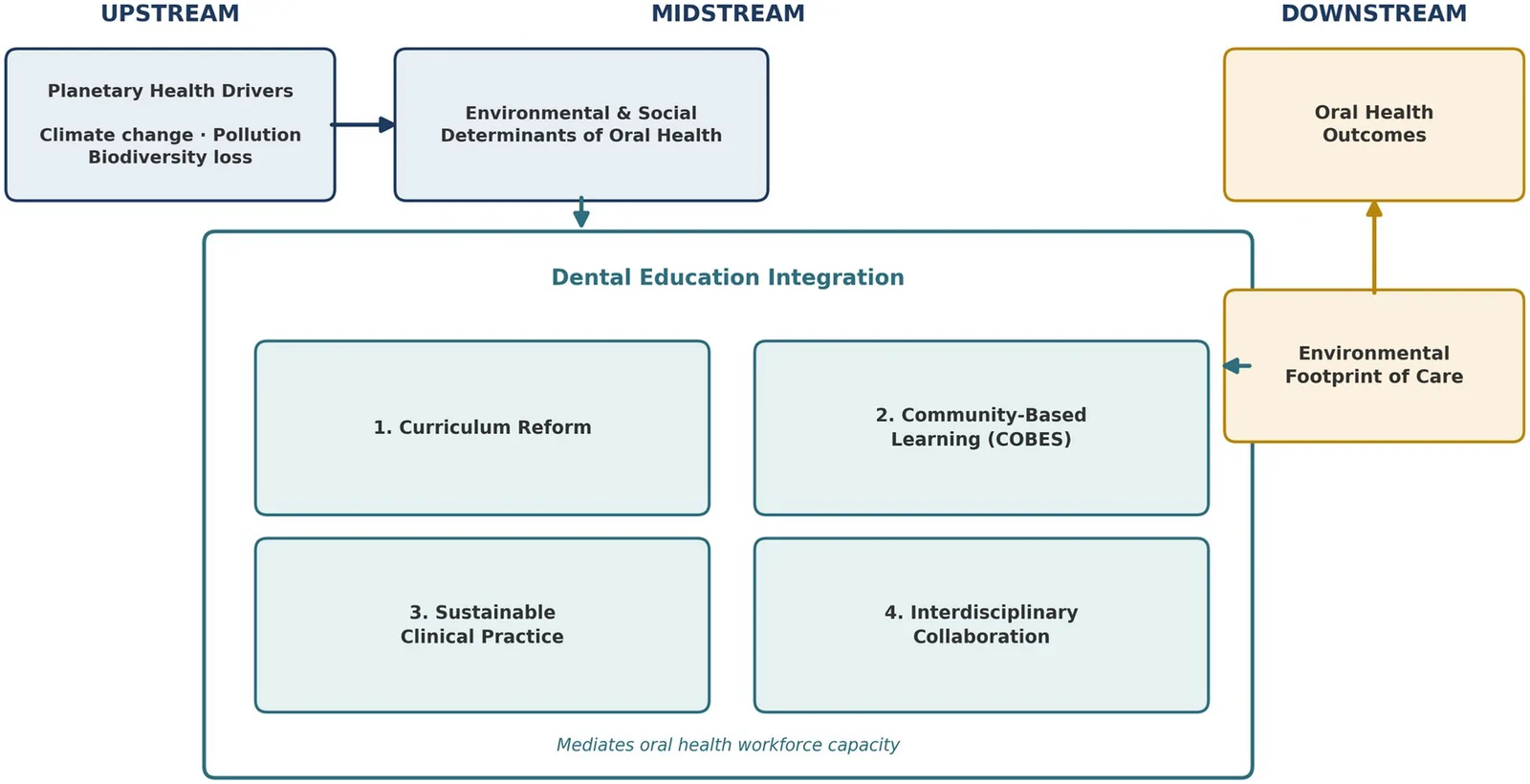
Proposed conceptual framework for embedding planetary health in dental education, tracing the upstream-to-downstream pathway from planetary health drivers to oral health and environmental outcomes, as mediated by the four-component dental education integration model.

The proposed framework operates across three levels: upstream, midstream, and downstream. At the upstream level, planetary health drivers including climate change, pollution, and biodiversity loss interact with environmental and social determinants to shape oral health outcomes. At the midstream level, dental education integration serves as the mediating mechanism through which workforce capacity is formed and transformed. Downstream, the framework aims to shift both oral health outcomes and the environmental footprint of oral healthcare delivery. Figure 1 illustrates these relationships and the four framework components through which educational integration is achieved.

## 7 Implications for global health education and policy

Integrating planetary health into dental education positions the profession within the most consequential global health priorities of our era. The Sustainable Development Goals—particularly SDG 3, SDG 4, SDG 6, SDG 12, SDG 13, and SDG 17—collectively establish a normative framework that includes oral health systems as active participants in sustainable development (14, 37). Dental educators can no longer treat these commitments as someone else's responsibility.

The WHO Global Oral Health Action Plan 2023–2030 explicitly calls for strengthening the oral health workforce, promoting prevention-oriented care models, and embedding oral health within universal health coverage frameworks (38). The framework in this paper provides a practical mechanism for operationalising these commitments at the level of professional training. Fisher et al. (19) have further argued that advancing global oral health must explicitly integrate planetary and One Health perspectives, and that this integration must be reflected in how oral health professionals are trained.

The 2021 Association of Medical Education in Europe (AMEE) Consensus Statement on Planetary Health and Education for Sustainable Healthcare marked a global milestone in health professional education (39). It recognises the agency of health professionals to protect the planetary foundations of health and calls for collective, transdisciplinary action, with a timeline for change linked to the SDGs by 2030. Our framework extends this agenda explicitly to the dental profession in the LMIC context, where it has the most urgent application and the greatest potential for systems-level impact.

The framework also strengthens oral health workforce resilience, a central concern in the Frontiers Research Topic to which this manuscript contributes. Dental professionals trained to understand environmental determinants, operate sustainably, and engage with communities are better equipped to respond to health emergencies, adapt clinical practice under disruption, and advocate for the inclusion of oral health in national emergency preparedness plans. These are competencies that current training largely does not build, and that the proposed model directly addresses (48).

## 8 Discussion

This manuscript contributes to the emerging discourse on planetary health in dental education, an area that has received limited scholarly and policy attention until recently (8, 40). The evidence base for this integration has grown considerably in recent years and now offers not only a rationale but also a practical road map. Duane et al. (28) provided the first systematic mapping of current practice in embedding environmental sustainability within dental curricula. Field et al. (29) established a pan-European competency framework and specific learning outcomes that this framework builds upon and extends into the LMIC context.

Our framework is distinguished from existing models in several ways. First, it is grounded in an African, LMIC institutional context: the COBES programme at Moi University. Prior work in this area has been conducted predominantly in European and North American settings. Second, this framework foregrounds community engagement as a pedagogical mechanism for sustainability integration, rather than treating curriculum design and assessment as the only levers of change. Third, it directly addresses the well-documented barriers to interdisciplinary collaboration in sub-Saharan African health education (35, 36), rather than treating interdisciplinarity as self-evidently achievable.

The evidence base for effective sustainability teaching has matured considerably. Dixon et al. (31) provided compelling evidence from a longitudinal curriculum integration study, demonstrating that sustained, structured embedding of environmental sustainability across all years of a dental programme produces significant and measurable improvements in student awareness, attitudes, and knowledge. At the level of targeted interventions, Baird et al. (32) demonstrated that interactive, outcome-aligned workshop formats can achieve significant gains within a single academic contact period. The Bamedhaf et al. (33) scoping review confirmed these findings globally: Structured, multi-modal strategies consistently outperform unstructured approaches. The approach to teaching sustainability matters as much as the decision to include it.

Several implementation challenges are worth acknowledging. Curriculum reform in health professional education is often slow, contested, and resource-intensive. Faculty capacity to deliver planetary health content is limited across most African dental schools, and institutional resistance to non-clinical additions is common. Addressing these barriers requires deliberate stakeholder engagement at institutional, national, and regional levels; targeted faculty development; and explicit alignment with accreditation standards and national health workforce policies (41). Tokenistic integration must be actively resisted. Planetary health competencies must feature in clinical assessments, community placement rubrics, and graduation requirements, not merely in elective lectures.

Future research should evaluate the impact of planetary health integration on student competencies, clinical practices, and community oral health outcomes, particularly within LMIC and African contexts. The growing international evidence base developed predominantly in Europe and North America has yet to be tested and adapted in these settings. Implementation science evaluations of scaled COBES adaptations, longitudinal cohort studies tracking graduates of reformed curricula, and qualitative inquiry into student and faculty perspectives would all contribute important evidence (42).

## 9 Conclusion

Integrating planetary health into dental education is not a fringe aspiration. It is a necessary paradigm shift towards sustainable, resilient, and equity-oriented oral health systems. The clinical and environmental challenges of this century demand oral health professionals who are not only technically capable but systemically literate, prevention-focused, and capable of contributing to a climate-resilient health sector. Dental education must rise to meet that demand.

Community-engaged training models such as COBES provide a practical and scalable platform for embedding sustainability, systems thinking, and prevention into dental curricula. The four-component framework in this manuscript spans curriculum reform, community-based learning, sustainable clinical practice, and interdisciplinary collaboration. It is explicitly situated in relation to existing international models and consensus frameworks (28, 29, 31, 39, 43), extending them into the LMIC and African community-engaged training context where they have not previously been applied.

Kenya sits at the intersection of high oral disease burden, environmental vulnerability, and a strong tradition of community-oriented health education. That positioning makes it both a learning laboratory and a model for broader adoption across LMICs. This framework offers a genuine pathway for oral health education to produce a future-ready workforce and contribute meaningfully to the global agenda of oral health within planetary boundaries.

## Data Availability

The original contributions presented in the study are included in the article/Supplementary Material, further inquiries can be directed to the corresponding author.
